# Antioxidant activity and mechanism of inhibitory action of gentisic and α-resorcylic acids

**DOI:** 10.1038/s41598-020-76620-2

**Published:** 2020-11-10

**Authors:** Azadeh Mardani-Ghahfarokhi, Reza Farhoosh

**Affiliations:** Department of Food Science and Technology, Faculty of Agriculture, Ferdowsi University of Mashhad, P.O. Box, 91775-1163 Mashhad, Iran

**Keywords:** Biochemistry, Chemistry

## Abstract

The antioxidant activity of gentisic (GA) and α-resorcylic (α-RA) acids was investigated by considering their molecular structures in various oxidative environments, including DPPH^·^ scavenging assay, stripped olive and soybean oils, and the corresponding oil-in-water emulsions. The mechanism of action in the oils was evaluated in the presence of different concentrations of the antioxidants at 60 °C, using the kinetic parameters the stabilization factor (*F*), the oxidation rate ratio (ORR), the activity (*A*), and the average rate of antioxidant consumption ($\overline{r}_{{{\text{AH}}}}$). GA was significantly more potent antioxidant than α-RA in all the environments. Although the less polar α-RA showed better activity in the emulsions rather than in the bulk oils, GA with an *ortho*-hydroxy structure had higher capacity to scavenge DPPH^·^, and LOO^·^ in the oils and emulsions. The lower performance of α-RA was attributed to its participation in side reactions of chain initiation (AH + LOOH → A^·^ + L^·^ + H_2_O) and propagation (A^·^ + LH → AH + L^·^) as competed with the main chain termination reaction (LOO^·^ + AH → LOOH + A^·^).

## Introduction

Lipid oxidation is the oxidative deterioration of unsaturated fatty acids via an autocatalytic radical chain process leading to adverse effects on both sensory and nutritional qualities of lipid systems. Antioxidants are one of the most important defense means to delay the reaction by scavenging chain-propagating peroxyl radicals. In recent years, interest in natural antioxidants has been increased due to the reported toxicologically negative effects of synthetic ones^1^.

Phenolic compounds are the major types of natural antioxidants that are widely found in plant sources and vegetable extracts. Antioxidant activity of phenolic acids in lipids has been investigated in so many studies to date. The capacity of phenolic acids to prevent lipid oxidation is undoubtedly related to their structural characteristics, especially the number of functional groups of higher efficacy^2^. Hydroxyl groups are considered among the substituents with very high electron-donating trait in phenolic compounds. Beside the number, the position on phenolic rings as well as intramolecular hydrogen bonding play important roles in their antioxidative performance. *Ortho* and *para*-hydroxyl groups are established to be the most effective positions, respectively^3,4^.

**Figure 1:**
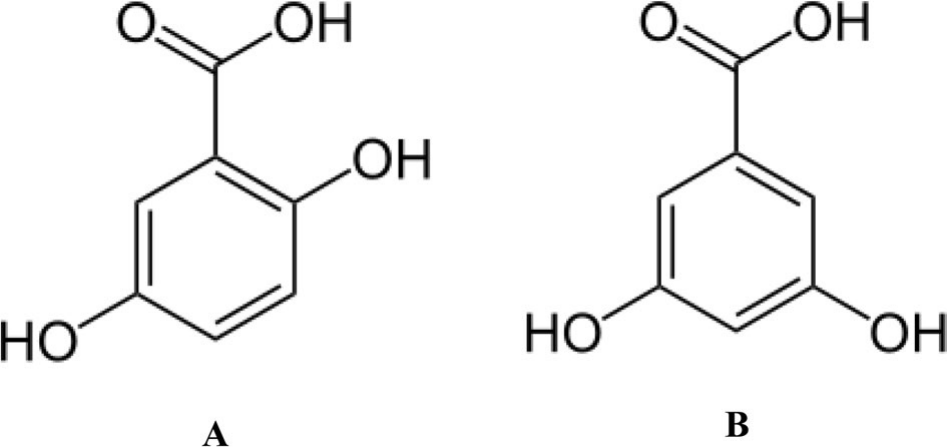
Chemical structure of gentisic acid (2,5-dihydroxybenzoic acid) (**A**) and α-resorcylic acid (3,5-dihydroxybenzoic acid) (**B**).

Gentisic (2,5-dihydroxybenzoic acid, GA, Fig. 1) and α-resorcylic (3,5-dihydroxybenzoic acid, α-RA, Fig. 1) acids are two dihydroxybenzoic acids present in many natural sources such as citrus fruits (*Citrus* spp.), grapes (*Vitis vinifera*), jerusalem artichoke (*Helianthus tuberosus*), sesame (*Sesamum indicum*), gentians (*Gentiana* spp.), red sandalwood (*Pterocarpus santalinus*), rose gum (*Eucalyptus grandis*), saxifrage (*Saxifraga* spp.), olive (*Olea europaea*), peanuts (*Arachis hypogaea*), chickpeas (*Cicer arietinum*), and hill raspberry (*Rubus niveus*)^5,6^. They are widely used in pharmaceutical industry and have been reported to show anxiolytic, antirheumatic, anticarcinogenetic, anti-inflammatory, and antimutagenic properties^7^. A recent study has established that these compounds are the fibroblast growth factor (FGF) inhibitors^2,8^.

The same type and number of substituents are attached on the aromatic rings of GA and α-RA. However, GA has generally been shown to possess higher activity in scavenging free radicals compared to α-RA^4^. Literature shows no information on their kinetics and mechanism of action. Hence, the aim of the present study was to investigate the relationship between the structural properties and mechanism of action of GA and α-RA in various oxidative environments, including DPPH^·^ scavenging assay as a fast and common test for radical scavenging activity of phenolic compounds, stripped olive and soybean oils, and the corresponding oil-in-water emulsions.

## Materials and methods

### Materials

GA and α-RA were purchased from Sigma-Aldrich (St. Louis, MO). All other chemicals and solvents used in this study were of analytical reagent grade and were provided by Sigma-Aldrich (St. Louis, MO) and Merck (Darmstadt, Germany). Refined soybean and olive oils with no antioxidant added were supplied by Roghan Nahan Gol (Shahre Kord, Chaharmahal and Bakhtiari province) and Etka (Mashhad, Khorasan Razavi province), respectively, and stored at −18 °C until analysis. The fatty acid compositions of the oils are shown in Table 1.

**Table 1:** Fatty acid composition of the vegetable oils.

	Olive oil	Soybean oil
**Fatty acid (%)**		
14:0	0.10 ± 0.01^b^	0.88 ± 0.04^a^
16:0	12.12 ± 0.27^a^	11.08 ± 0.07^b^
16:1	1.33 ± 0.04^a^	0.81 ± 0.01^b^
17:0	3.98 ± 0.10^a^	0.03 ± 0.00^b^
18:0	4.30 ± 0.09^b^	4.74 ± 0.11^a^
18:1	69.40 ± 0.19^a^	22.18 ± 0.12^b^
18:2	8.77 ± 0.17^b^	51.12 ± 0.68^a^
18:3	0.62 ± 0.06^b^	8.01 ± 0.06^a^
20:0	0.64 ± 0.01^a^	0.42 ± 0.06^b^
20:1	0.31 ± 0.05^a^	–
22:0	0.26 ± 0.08^a^	–
Others	0.37 ± 0.11^b^	0.73 ± 0.01^a^

Means ± SD (standard deviation) within a row with the same lowercase letters are not significantly different at *p* < 0.05.

### DPPH^·^ scavenging activity assay

2,2-Diphenyl-1-picrylhydrazyl (DPPH) radical scavenging ability of the antioxidants was measured according to Lima et al.^9^. The samples were reacted with the stable DPPH^·^ in methanol. The absorbance of the samples was read against a blank at 517 nm after 30 min at room temperature in the dark. Inhibition of free DPPH^·^ in percent (I%) was calculated as follows:

1 $${\text{I\% = }}\frac{{{\text{A}}_{{{\text{Blank}}}} - {\text{A}}_{{{\text{Sample}}}} }}{{{\text{A}}_{{{\text{Blank}}}} }} \times {100}$$

where A_Sample_ is the absorbance of the test compound and A_Blank_ is the absorbance of the control reaction (containing all reagents except the test compound). The concentration of sample required for 50% inhibition of DPPH^·^ (IC_50_ value) was calculated by linear regression analysis of dose–response curve plotting between the I% and concentrations. Antioxidant reducing power (ARP) was calculated from the IC_50_ value as follows:

2 $${\text{ARP = }}\frac{1}{{{\text{IC}}_{{{50}}} }} \times {100}$$

where the larger the ARP value, the more efficient the antioxidant.

### Partition coefficient (log P)

Solutions (0.3 mM) of each compound in 1-octanol were kept at 60 °C for 1 h. The maximum absorbance was read by UV spectrum (A_0_). Equal volumes of this solution and acetate buffer (0.1 M, pHs 3.5 and 5.5) were vortexed for 1 min. The UV spectrum of the 1-octanol layer was determined after 30 min (A_x_). The partition coefficient (log P) of antioxidant was calculated according to the following equation^10^:

3 $${\text{P = }}\frac{{{\text{A}}_{{\text{x}}} }}{{{\text{A}}_{{0}} - {\text{A}}_{{\text{x}}} }}$$

### Stripping the oils

The purified triacylglycerols of soybean and olive oils were obtained by removing indigenous antioxidants by adsorption chromatography^11^: 130 g of oils were purified twice by passing in a glass column (25 × 2.5 cm i.d), packed with 70 g of aluminum oxide 60 activated at 200 °C for 3 h in bottom layer, and 15 g of silica gel activated at 160 °C for 3 h in upper layer. Triacylglycerols were drawn in the dark through the column by suction without solvent. The samples were stored at − 20 °C in an inert atmosphere. The purified oils were contained inconsiderable amounts of hydroperoxides (peroxide value < 1 meq kg^−1^)^12^, phenolics^13^ and tocopherols (< 1 mg kg^−1^)^14^.

### Preparation and oxidation of the oils and oil-in-water emulsions

Soybean and olive oils containing different concentrations of the antioxidants (0.02, 0.04, 0.08, 0.16, and 0.32% of GA and α-RA) were prepared by adding aliquots of their solutions in acetone. The solvent were removed under nitrogen. Oxidation was performed in the dark at 60 °C. One-mm layers of the prepared oils (4 g) were oxidized in a Petri dish having a diameter of 9 cm. Under these conditions, the process took place in a kinetic regime, i.e., at a sufficiently high oxygen concentration which the diffusion rate does not influence the oxidation rate^15^. Three replications of the samples were stored.

The aqueous phase of emulsion was prepared by dispersing Tween 20 in distilled water, followed by stirring at room temperature overnight to ensure complete dispersion and hydration. The oil-in-water emulsion was prepared by adding 10 wt% of the stripped oils containing 0.02% of the antioxidants to 90 wt% of the aqueous phase at ambient temperature and homogenized for 2 min in a high-speed blender (Waring Commercial, USA). To obtain a stable emulsion, the mixture was vortexed by ultra-Turrax (3 min, ~ 12,000 rpm). During each pass, the emulsions were collected in a beaker kept in an ice bath. Emulsion samples were kept in an oven at 60 °C. Oil extraction from the emulsions for analysis was carried out by shaking a mixture of a methanol/chloroform solvent system (1:1 v/v) and the emulsion for 1 min and then centrifuging for 1 min at 700×*g*. The lower lipid layer was collected and its solvent evaporated using a stream of nitrogen. The oxidation process was followed by withdrawing samples at certain time intervals and subjecting them to spectrophotometric determination of the peroxide value (PV) as primary oxidation products. Kinetic curves of peroxide accumulation were plotted. The x-coordinate of intersection point of two straight lines fitted on the initiation and propagation stages of the oil oxidation was calculaed as induction period (IP)^16^.

### PV measurement

The vegetable oil samples (≤ 0.01–0.30 g) were added to a glass tube containing 9.8 mL chloroform–methanol (7:3 v/v) and were vortexed for 2–4 s. 50 mL of ammonium thiocyanate solution (30% w/v) was added the sample was mixed on a vortex mixer for 2–4 s. Then iron (II) chloride solution [50 mL, (0.4 g barium chloride dehydrate dissolved in 50 mL H_2_O) + (0.5 g FeSO_4_·7 H_2_O dissolved in 50 mL H_2_O) + 2 mL 10 M HCl], with the precipitate, barium sulfate, filtered off to produce a clear solution]) were added, and the sample was vortexed for 2–4 s. After 5 min incubation at room temperature, the absorbance of the sample was read against a blank sample (containing all the reagents except the sample) at 500 nm (UV–Vis spectrophotometer, Model 160A Shimadzu, Kyoto, Japan). Results were reported as milliequivalents of oxygen per kilogram of oil^12^.

### Kinetic parameters and mechanism of action

The inhibitory action of the antioxidants (AH) in the oil samples was described by the stabilization factor *F*, the oxidation rate ratio ORR, the activity *A*, and the average rate of antioxidant consumption $\overline{r}_{{{\text{AH}}}}$^17^. The parameter *F* is a measure of effectiveness, representing the possibility of blocking the chain radical process by scavenging peroxide radicals:

4 $$F = \frac{{{\text{IP}}_{{{\text{AH}}}} }}{{{\text{IP}}_{{0}} }}$$

where IP_AH_ is the induction period in the presence of antioxidant, and IP_0_ is the induction period in the absence of antioxidant. ORR is an inverse measure of antioxidant strength (the lower the ORR, the stronger the inhibitor).

5 $${\text{ORR = }}\frac{{W_{{{\text{AH}}}} }}{{W_{{0}} }}$$

where *W*_AH_ and *W*_0_ are the oxidation rate in the presence and absence of an antioxidant, respectively. The parameter *A* is a general parameter unifying the parameters *F* and ORR:

6 $$A = \frac{F}{{{\text{ORR}}}}$$

The parameter $\overline{r}_{{{\text{AH}}}}$ was calculated by the following formula:

7 $$\overline{r}_{{{\text{AH}}}} = \frac{{{\text{[AH]}}_{{0}} }}{{{\text{IP}}_{{{\text{AH}}}} }}$$

where [AH]_0_ is the initial concentration of the antioxidant.

**Scheme 1:**
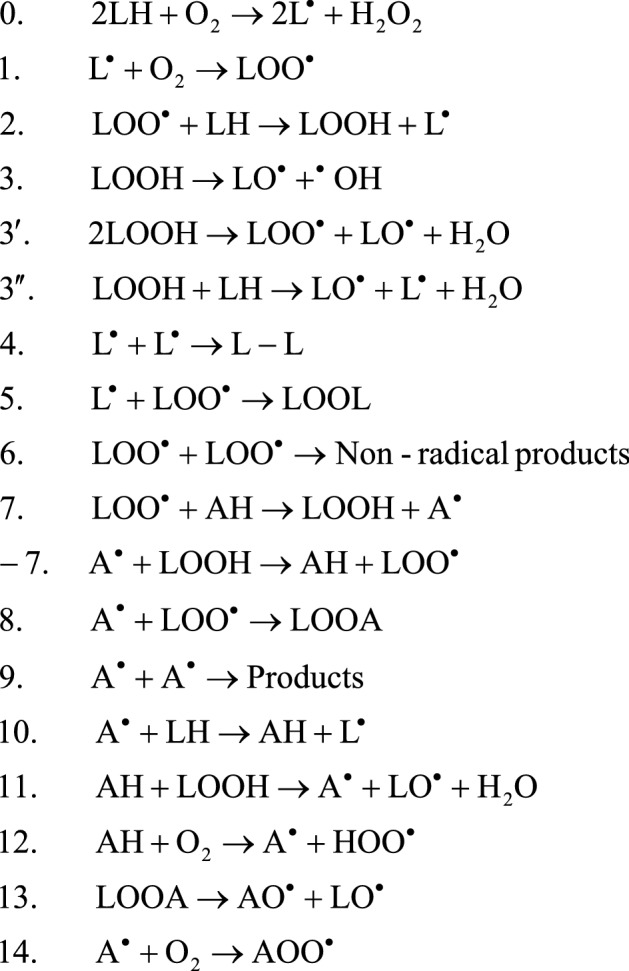
Non-inhibited (0–6) and inhibited (7–14) oxidation reactions. LH, oxidizable lipid substrate; L^·^, lipid radical; LOOH, lipid hydroperoxide; LOO^·^, peroxyl radical; LO^·^, alkoxyl radical; AH, antioxidant molecule; A^·^, antioxidant radical^18^.

The mechanism of inhibitory action of GA and α-RA was determined based on the participation of their antioxidant molecules (AH) and radicals (A^·^) in a series of reactions (Scheme 1)^18^. The possibility of blocking the chain radical process through the interaction with peroxide radicals (the main reaction of chain termination 7) is represented by the parameter *F*. Linear dependence of the factor *F* on antioxidant concentration illustrates the participation of antioxidant molecule mainly in reaction 7. The absence of linearity of this dependence has been due to the participation of antioxidant molecule in reactions other than the main reaction of chain termination 7, namely reaction 11 and/or 12. In this case, the Eq. (8) provides a possibility to identify the occurrence of these two side reactions^15^.

8 $$\overline{r}_{{{\text{AH}}}} = K_{eff} [{\text{AH]}}^{n} + \frac{{W_{i} }}{f}$$

where *K*_eff_ is the rate constant of antioxidant consumption in side reaction(s) of chain initiation and dependeds on the character of lipid substrate, *W*_*i*_ (M s^−1^) is the average rate of initiation during IP, and *f* is the stoichiometric coefficient of inhibition, determining the number of peroxide radicals scavenged by an antioxidant molecule. If antioxidant molecule does not take part in any side reaction(s), the order rate side reaction will be zero (*n* = 0). A linear dependence at* n* = 1 means that antioxidant molecule participates in only one side reaction of chain initiation (reactions 11 or 12). A second order rate side reaction (*n* = 2) denotes the participation of antioxidant in the both side reactions.

In order to thoroughly evaluate the inhibitory capacity of an antioxidant it is necessary to see if the radical of antioxidant (A^·^) takes part in chain propagation reactions − 7, 10, and 14:

9 $$W_{{{\text{AH}}}} \approx [{\text{AH]}}^{n}$$

Previous investigations on inhibitory effect of antioxidants indicated that linear dependence at *n* =  − 1 means that antioxidant radical does not take part in chain propagation reactions while if A^·^ participates in one reaction of chain propagation, linear dependence at *n* =  − 0.5 is observed^19^. It has been demonstrated that the reaction 10 should be this reaction^20^. Nonlinear dependence at both *n* =  − 1 and *n* =  − 0.5 indicates that the antioxidant radicals were involved in more than one reaction of chain propagation. Lack of dependency (*n* = 0) means that antioxidant molecule is so active that peroxide radicals (LOO^·^) react faster with antioxidant than with lipid substrate (LH)^19^.

### Statistical analysis

All determinations were carried out in triplicate and data were subjected to analysis of variance (ANOVA). ANOVA and regression analyses were performed according to SPSS and Excel software. Significant differences between means were determined by Duncan’s multiple range tests. The p values less than 0.05 were considered statistically significant.

## Results and discussion

### Partition coefficient (log P)

Partitioning between the 1-octanol and aqueous phases depends on the polarity and structural properties of the antioxidants. More hydrophilic structures tend to move towards aqueous phases, being shown by lower values of log P. Regardless of the level of pH, GA showed lower values of log P compared to α-RA (Table 2), implying its molecular structure of reduced symmetry due to the polar functional groups located in two opposite sides of the aromatic ring.

**Table 2:** Partition coefficient (log P) and antioxidant reducing power (ARP, 100/IC_50_) of gentisic (GA) and α-resorcylic acids (α-RA).

Compound	log P at pH		ARP
	3.5	5.5	
GA	0.42 ± 0.02^b^	− 0.51 ± 0.04^a^	2.7 ± 0.04^a^
α-RA	0.63 ± 0.01^a^	− 0.24 ± 0.02^b^	0.26 ± 0.01^b^
GA/α-RA ratio	0.67	2.13	10.4

Means ± SD (standard deviation) within a column with the same lowercase letters are not significantly different at *p* < 0.05.

**Figure 2:**
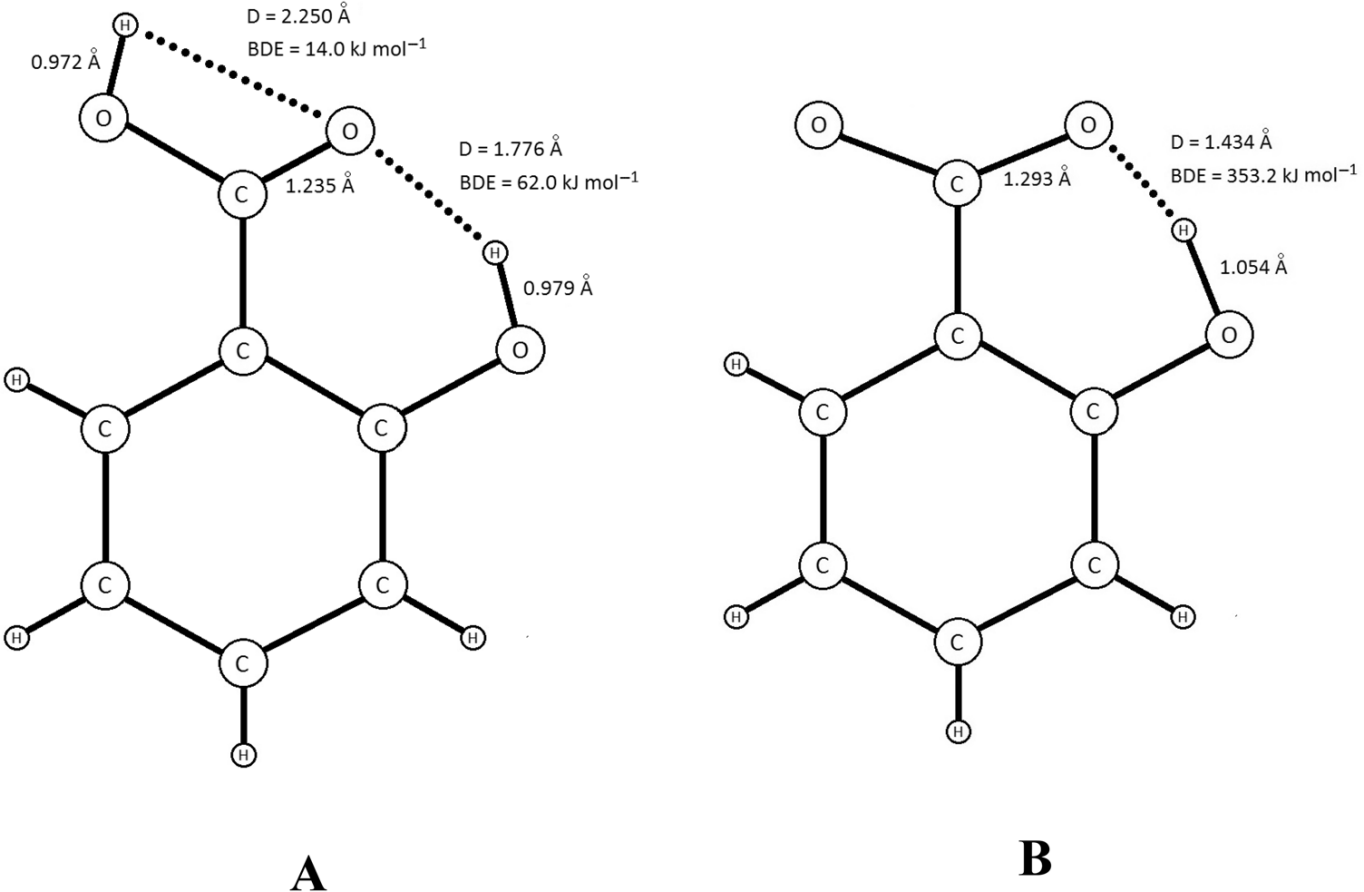
The predominant hydrogen bonding conformer of salicylic acid (2-hydroxybenzoic acid (**A**) and its carboxylate anion, salicylate (**B**).

The higher value of pH made the antioxidants more hydrophilic. This can clearly be attributed to the deprotonation of the carboxyl groups with pK_a_ values of 2.97 and 4.04 at 25 °C for GA and α-RA, respectively. The ionization of carboxyl groups is suppressed at pK_a_ − 1 and is complete at pK_a_ + 1^21^. However, the level of polarity increased more in GA than in α-RA at the higher pH value, so that the GA/α-RA polarity ratio increased from 0.67 at pH 3.5 to 2.13 at pH 5.5 (Table 2). This may be explained by the analogy between the intramolecular hydrogen bonding of the vicinal hydroxyl and carboxyl groups in gentisic and salicylic acids. The chemical structure A illustrated in Fig. 2 has been shown to be the predominant hydrogen bonding conformer of salicylic acid among a number of possible conformers that involve the other oxygen and hydrogen atoms in the adjacent hydroxyl and carboxyl groups^22^. Concerning the MP2/6-31G** method of calculating the bond energies, geometries, and frequencies, Chen and Shyu^23^ showed a quite shorter and, therefore, stronger intramolecular hydrogen bond in salicyliate than in the parent molecule^23^ (Fig. 2). This was consistent with the higher atomic charge for the oxygen and hydrogen atoms, leading naturally to a higher dipole moment or polarity in the salicylic acid anion.

### DPPH^·^ scavenging activity

**Figure 3:**
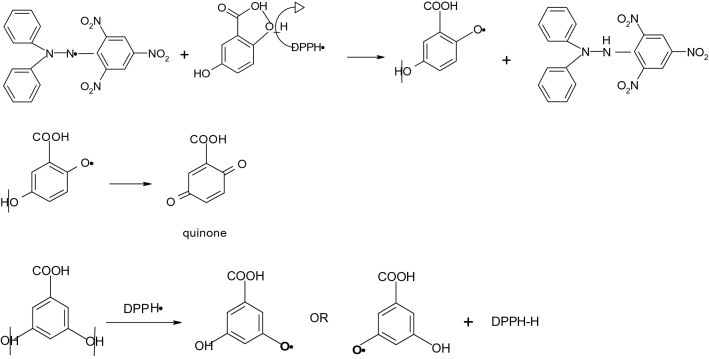
Possible DPPH radical scavenging mechanism of gentisic and α-resorcylic acids and formation of the corresponding quinone and phenoxyl radicals.

DPPH^·^ scavenging assay is the most frequently used method which offers the first approach for evaluating antioxidant activity of various natural and synthetic compounds. Antioxidants can quench DPPH^·^ by electron donation or hydrogen atom transfer and disappear the purple color of the DPPH^·^ solution measured at 517 nm. As shown in Table 2, GA was found to be ~ 10 times more effective than α-RA in scavenging DPPH^·^. This was in accordance with the findings of Sroka and Cisowski^24^ who showed GA to be of higher antiradical capacity than α-RA. This can be due to the higher stoichiometric inhibition factor of GA, so that following the first hydrogen abstraction (Fig. 3), it forms a stable semiquinone resonance hybrid that undergoes a second hydrogen abstraction, producing an *ortho* benzoquinone structure. α-RA, however, results only in semiquinone radical intermediates with moderate resonance delocalization^25^. Moreover, it would be expected that the more polarity and solubility of GA improves its molecular mobility and ability to scavenge DPPH^·^.

### Antioxidant activity in the bulk oils

**Figure 4:**
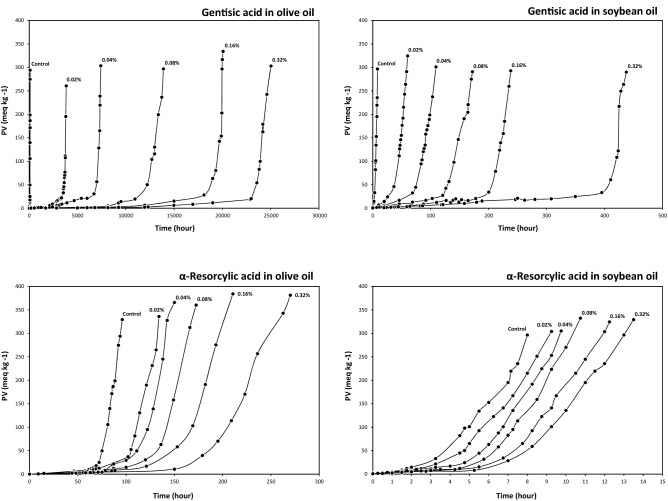
Kinetic curves of hydroperoxide accumulation during the oxidation of olive and soybean oils at 60 °C in the absence (control) and presence of different concentrations of gentisic and α-resorcylic acids.

The kinetic curves of hydroperoxide accumulation during the oxidation of olive and soybean oils in the absence and presence of the different concentrations of GA and α-RA are illustrated in Fig. 4. The extended IPs were observed with increasing in the antioxidants concentration.

**Figure 5:**
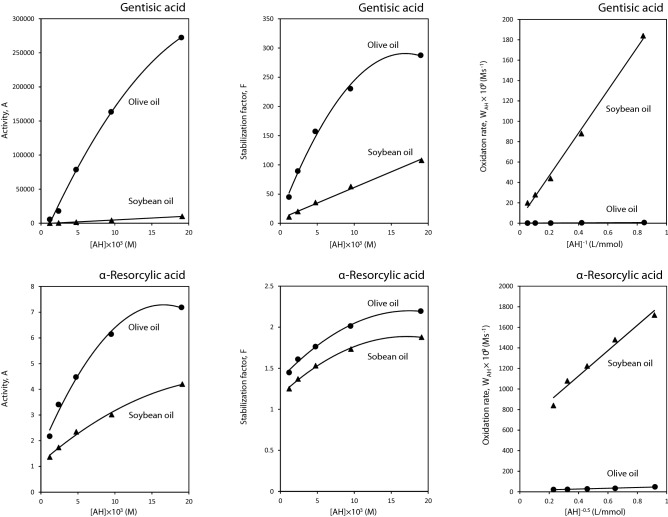
Concentration dependence of the antioxidant activity, stabilization factor, and oxidation rate of gentisic and α-resorcylic acids in olive and soybean oils at 60 °C.

The higher antioxidative potency of GA than α-RA in the chemical environment of the DPPH^·^ scavenging assay (Table 2) was dramatically promoted in the lipid systems experimented. Considering the values of the kinetic parameters *A* and $\overline{r}_{{{\text{AH}}}}$ (Fig. 5 and Table 3), GA showed antioxidaive performances incomparably better than α-RA in the two bulk phase oils. The antioxidant activities were significantly higher in the olive oil of considerably lower degree of unsaturation (Table 1). The relative reaction rate of oxygen with oleate, linoleate, and linolenate has been reported to be 1, 27, and 77, respectively^26^. The hyperbolic patterns of antioxidant activity with concentration (Fig. 5) has been attributed to more significantly elevated levels of the pro-oxidant activity of antioxidants at higher concentrations^27^.

**Table 3:** The kinetic parameters of hydroperoxide formation during the oxidation of olive and soybean oils containing five concentrations of gentisic (GA) and α-resorcylic (α-RA) acids at 60 °C.

	[AH] (%)	[AH] (× 10^3^) (M)	*F*	ORR	*A*	*W*_AH_ (× 10^9^) (M s^−^^1^)	$\overline{r}_{{{\text{AH}}}}$ (× 10^10^) (M s^−^^1^)
**Bulk phase olive oil**							
GA	0	0	–	–	–	72.0 ± 4.0^i^	–
	0.02	1.18	45.0 ± 4.0^g^	0.008 ± 0.000^n^	5625 ± 16^f^	0.57 ± 0.02^o^	0.91 ± 0.04^r^
	0.04	2.37	89.0 ± 9.0^e^	0.005 ± 0.000^o^	17,875 ± 19^d^	0.36 ± 0.04^p^	0.92 ± 0.02^r^
	0.08	4.74	157 ± 12^c^	0.002 ± 0.000^p^	78,668 ± 23^c^	0.14 ± 0.01^q^	1.04 ± 0.03^q^
	0.16	9.49	230 ± 19^b^	0.001 ± 0.000^q^	163,439 ± 36^b^	0.10 ± 0.01^r^	1.42 ± 0.05^p^
	0.32	18.99	287 ± 25^a^	0.001 ± 0.000^q^	272,415 ± 46^a^	0.07 ± 0.00^s^	2.29 ± 0.04^o^
α-RA	0.02	1.18	1.44 ± 0.02^q^	0.66 ± 0.02^c^	2.17 ± 0.15^q^	48.6 ± 0.1^h^	28.3 ± 3.9^n^
	0.04	2.37	1.61 ± 0.03^o^	0.47 ± 0.02^e^	3.41 ± 0.14^n^	34.6 ± 0.1^j^	51.1 ± 4.3^m^
	0.08	4.74	1.76 ± 0.02^n^	0.39 ± 0.02^g^	4.47 ± 0.22^m^	28.3 ± 0.9^k^	93.3 ± 2.1^k^
	0.16	9.49	2.01 ± 0.02^l^	0.32 ± 0.02^h^	6.14 ± 0.18^l^	23.6 ± 0.8^l^	164 ± 12^g^
	0.32	18.99	2.19 ± 0.03^k^	0.30 ± 0.01^h^	7.18 ± 0.22^k^	22.1 ± 0.1^m^	301 ± 21^f^
**Bulk phase soybean oil**							
GA	0	0	–	–	–	1880 ± 21^a^	–
	0.02	1.19	11.0 ± 0.2^j^	0.097 ± 0.001^i^	112 ± 1^j^	184 ± 11^f^	86.9 ± 2.1^l^
	0.04	2.38	20.2 ± 0^i^	0.046 ± 0.002^j^	432 ± 3^i^	88.0 ± 9.0^g^	94.4 ± 4.1^k^
	0.08	4.77	35.5 ± 1.9^h^	0.023 ± 0.001^k^	1519 ± 5^h^	44.0 ± 3.0^i^	108 ± 8^j^
	0.16	9.55	63.0 ± 2.8^f^	0.014 ± 0.002^l^	4230 ± 8^g^	28.0 ± 2.0^k^	122 ± 3^i^
	0.32	19.10	108 ± 4^d^	0.010 ± 0.001^m^	10,133 ± 9^e^	20.0 ± 1.0^n^	142 ± 2^h^
α-RA	0.02	1.19	1.25 ± 0.01^s^	0.91 ± 0.04^a^	1.36 ± 0.07^s^	1720 ± 26^b^	763 ± 20^e^
	0.04	2.38	1.36 ± 0.03^r^	0.78 ± 0.01^b^	1.74 ± 0.04^r^	1480 ± 41^c^	1394 ± 45^d^
	0.08	4.77	1.53 ± 0.02^p^	0.65 ± 0.03^c^	2.35 ± 0.01^p^	1224 ± 23^d^	2500 ± 58^c^
	0.16	9.55	1.73 ± 0.03^n^	0.57 ± 0.02^d^	3.01 ± 0.06^o^	1080 ± 52^d^	4421 ± 44^b^
	0.32	19.10	1.87 ± 0.04^m^	0.44 ± 0.01^f^	4.20 ± 0.09^m^	840 ± 11^e^	8162 ± 29^a^

Means ± SD (standard deviation) within a column with the same lowercase letters are not significantly different at *p* < 0.05. Olive oil: IP_0_ = 80.0 h, *W*_0_ = 72.0 × 10^−7^ M s^−1^; soybean oil: IP_0_ = 3.46 h, *W*_0_ = 18.80 × 10^−7^ M s^−1^.

Kinetic evaluations can provide us with helpful information on the mechanism of action and, thereby, the different activities of the antioxidants in the bulk oils^15,17,19^. The concentration dependence of the stabilization factor *F* was linear only for the GA-inhibited soybean oil oxidation (Fig. 5), indicating the participation of the antioxidant molecules mainly in the reaction 7 (Scheme 1). The absence of linearity in the other treatments (Fig. 5) has been attributed to the participation of antioxidant molecules in the side reactions 11 and/or 12 in addition to the main reaction of chain termination 7 (Scheme 1). The concentration dependence of the average rate of GA and α-RA consumption (Eq. 8) in the olive and soybean oils was linear at *n* = 1. This means that the antioxidant molecules participate in one side reaction of chain initiation 11 or 12. The parameter *K*_eff_ has been shown to be dependent on the character of lipid system and its value is higher in more unsaturated substrates^28^. Our results in the present study (Table 4) were in accordance with this fact that the main side reaction of chain initiation in these cases should be the reaction 11, which depends on the hydroperoxide reactivity (Scheme 1).

**Table 4:** Kinetic parameters characterizing the rate constant of gentisic acid (GA) and α-resorcylic acid (α-RA) consumption in side reaction(s) of chain initiation, *K*_*eff*_, and the average rate of initiation, *W*_*i*_/*f*, during the oil samples oxidation at 60 °C.

Antioxidant	Lipid system	*K*_*eff*_ (× 10^7^) (s^−^^1^)	*W*_*i*_/*f* (× 10^10^) (M s^−^^1^)
GA	Olive oil	0.08 ± 0.01^d^	0.73 ± 0.03^d^
	Soybean oil	2.96 ± 0.01^c^	88.7 ± 2.7^b^
α-RA	Olive oil	15.11 ± 1.8^b^	16.2 ± 0.1^c^
	Soybean oil	409 ± 6^a^	425 ± 9^a^

Means ± SD (standard deviation) within a column with the same lowercase letters are not significantly different at *p* < 0.05.

As presented in Fig. 5, the concentration dependence of *W*_AH_ versus of GA in the bulk oils (Eq. 9) was linear at *n* =  − 1. This means that GA radicals do not take part in the side reactions of chain propagation (− 7, 10, and 14) in the both lipid systems. Processing the data obtained for α-RA showed linear dependences of *W*_AH_ versus the antioxidant concentration at *n* =  − 0.5. This signifies that α-RA radicals participate in one of the side reactions of chain propagation. As established in the previous studies by Yanishlieva and Marinova^20^, this reaction is the reaction 10.

### Antioxidant activity in the O/W emulsions

Oil-in-water emulsions are often more susceptible to oxidation than bulk phase oils due basically to their higher surface areas, promoting interaction of the oil with pro-oxidants in the aqueous phase^29^. Therefore, it is important to also include information on the effectiveness of an antioxidant in oil/water emulsion systems for a more comprehensive assessment of antioxidant activity.

As shown in Table 5, GA was still an oxidation inhibitor of remarkably higher activity than α-RA in the emulsions. Also, the antioxidants showed better performances in the olive than in soybean oil-in-water emulsions. However, GA and α-RA exerted the antioxidant activities lower and higher, respectively, in the emulsions than in the bulk oils (Tables 4, 5). The polar paradox theory states that more polar antioxidants are more effective in less polar media. In emulsion systems, polar antioxidants would tend to partition into the aqueous phase, where they would not be able to protect the lipid phase effectively^30^. In fact, the activity of antioxidants in dispersed systems relates to their radical scavenging activities as well as their affinity towards the water–oil interface, the site where oxidation occurs. Although the *meta*-hydroxy structure of α-RA could not be a strong radical scavenger like the *ortho*-hydroxy structure of GA, its less polar structure caused it to show better activity in the emulsions rather than in the bulk oils. In other words, α-RA was able to incorporate higher concentrations of its less polar molecules (Table 2) into the oil–water interfaces and to inhibit reactive lipid radicals.

**Table 5:** The kinetic parameters of hydroperoxide formation during the oxidation of the olive and soybean oil-in-water emulsions containing gentisic (GA) and α-resorcylic (α-RA) acids at 60 °C.

	[AH] (%)	[AH] (× 10^3^) (M)	*F*	ORR	*A*
**Olive oil emulsion**					
	0	0	–	–	–
GA	0.02	1.18	10.1 ± 0.2^a^	0.02 ± 0.01^c^	505 ± 3^a^
α-RA	0.02	1.18	2.19 ± 0.02^c^	0.10 ± 0.01^b^	21.7 ± 1.2^c^
**Soybean oil emulsion**					
	0	0	–	–	–
GA	0.02	1.19	5.51 ± 0.03^b^	0.13 ± 0.01^b^	40.6 ± 0.1^b^
α-RA	0.02	1.19	2.12 ± 0.01^d^	0.23 ± 0.01^a^	9.04 ± 0.10^d^

Means ± SD (standard deviation) within a column with the same lowercase letters are not significantly different at *p* < 0.05. Olive oil emulsion: IP_0_ = 12.3 h, *W*_0_ = 96.72 × 10^−7^ M s^−1^; soybean oil emulsion: IP_0_ = 0.93 h, *W*_0_ = 16.48 × 10^−7^ M s^−1^.

## Conclusions

In this study, the antioxidant activity and mechanism of action of gentisic and α-resorcylic acids were investigated. From a kinetic point of view, gentisic acid acted more effectively compared to α-resorcylic acid in various oxidative environments. Higher polarity/solubility and stoichiometric inhibition factor of gentisic acid improves its ability to scavenge DPPH^·^ in methanol and LOO^·^ in the bulk oils. Although the *meta-*hydroxy α-resorcylic acid showed low antioxidant activities in all the environments studied, incorporating higher concentrations of its less polar molecules caused it to show better activity in the emulsions than in the bulk oils. The mechanistic evaluations of antioxidant action showed better performance of gentisic acid in soybean oil, which was attributed to the participation of its molecules in the main reaction of chain termination 7 as competed with the side reactions of chain propagation 11 and 12.
